# Awareness and correlates of the role of physical activity in breast cancer prevention among Japanese women: results from an internet-based cross-sectional survey

**DOI:** 10.1186/1472-6874-14-80

**Published:** 2014-07-07

**Authors:** Rina Miyawaki, Ai Shibata, Kaori Ishii, Koichiro Oka

**Affiliations:** 1 Graduate School of Sport Sciences, Waseda University, Saitama, Japan; 2 Faculty of Health and Sport Sciences, University of Tsukuba, Ibaraki, Japan; 3 Faculty of Sport Sciences, Waseda University, Saitama, Japan

**Keywords:** Breast cancer, Physical activity, Cancer prevention, Health communication, Awareness, Correlates

## Abstract

**Background:**

Although considerable evidence has demonstrated that physical activity is associated with breast cancer prevention, few studies have assessed the level of awareness of this association. Awareness is a key first step to successful of behavior change. Increasing awareness may contribute to promote physical activity and prevent breast cancer at the population level. The present study examined the prevalence and correlates of awareness about the role of physical activity in breast cancer prevention among Japanese women.

**Methods:**

1,000 Japanese women aged 20–69 years (mean age: 44.3 ± 13.4 years) who responded to an internet-based cross-sectional survey. Awareness of the role of physical activity in breast cancer prevention, knowledge of breast cancer (symptom, risk factor, screening), exposure to information about physical activity and cancer, a self-reported physical activity, and sociodemographic variables (age, marital status, having a child, education level, employment status, and household income) were obtained. Force-entry logistic regression analysis was used.

**Results:**

The prevalence of awareness was 31.5% (95% CI: 28.6-34.4). Factors significantly associated with awareness included sociodemographic variables, exposure to information, and knowledge of breast cancer. Being married (AOR, 95% CI: 1.75, 1.05–2.92) was positively related to awareness, while having children (0.65, 0.36–0.86) was negatively related. College graduates or those with higher levels of education (1.50, 1.01–2.22) were significantly more likely to be aware than those who had not graduated high school. Moreover, exposure to information (2.11, 1.51–2.95), and high knowledge of symptoms (2.43, 1.75–3.36) were positively associated with awareness. Finally, low knowledge of risk factors (0.30, 0.22–0.40) was negatively associated with awareness.

**Conclusions:**

Japanese women through internet-based study were poorly aware of the role of physical activity in breast cancer prevention. Awareness was especially low among individuals with children and higher knowledge of risk factors whereas high in married women, those with higher educational level, exposure to information, and greater knowledge of symptoms. The findings suggest that strategies to increase the awareness about the preventive role of physical activity are needed for breast cancer prevention in consideration of subgroups with low awareness.

## Background

Breast cancer is the most common cancer among women [1]. The mortality and incidence rate of breast cancer has been continuously increasing. In Japan, breast cancer accounted for 8.6% of all cancer deaths in 2011 and 19.0% of new cases of cancer in 2007 [2]. A number of studies have consistently confirmed that lactation and low alcohol consumption can decrease the risk of breast cancer [3,4]. In addition, physical activity can decrease the risk of breast cancer [5] via several mechanisms such as a decrease in sex hormones and metabolic hormones, reduction of inflammation, and improvement of immune function [6]. Previous studies also revealed that physically active women could reduce their risk of breast cancer by approximately 20–30% compared with inactive women [7]. However, only 28.5% of Japanese women have been reported to engage in physical activity on a regular basis [8].

Awareness is the first key step to successful behavior change [9,10]. Raising awareness about the role of physical activity in breast cancer prevention could potentially lead to a change in knowledge, attitudes, intension, and promote physical activity in a stepwise process [11]. Previous studies found that exposure to information about the link between exercise and colon cancer increased exercise motivation [12,13]. Consequently, an increase in awareness about the role of physical activity in cancer prevention may promote physical activity for the reduction of cancer. Previously, only a small number of studies have examined awareness levels regarding the preventive role of physical activity on certain cancer. These studies reported that approximately 15% of adults in the United States [14], and 30% of adults across all 21 European countries [15] were aware of the effect of physical activity in colon cancer prevention. Among Japanese adults, the attributable fraction of physical activity was reported to be low (26%) for cancer-causing viral infection [16]. However, none of these studies have focused on breast cancer. For women, breast cancer may be a more serious concern than other cancer because of changes in bodily shape caused by having a mastectomy, and the associated feelings of loss, depression, and anxiety. Thus, awareness about the protective effect of physical activity against breast cancer may effectively motivate women to become more physically active. Furthermore, it may reduce population-wide breast cancer risk.

Better understanding of the correlates of awareness about the role of physical activity in breast cancer prevention is essential to identifying targeted individuals and developing tailored interventions. Recent research observed that awareness of the role of physical activity in colon cancer was positively related to higher educational level, engagement in physical activity, previous exposure to information about cancer and physical activity, and higher knowledge about cancer [14,17].

However, no study examined for breast cancer. Thus, the aim of the present study was to examine the prevalence and correlates of awareness about the role of physical activity in breast cancer prevention among Japanese women.

## Methods

### Sample

The participants were 1,000 Japanese women aged 20–69 years (44.3 ± 13.4 years) who responded to an internet-based cross-sectional survey conducted through a Japanese social research company (MyVoice Communication, Inc, Japan). This research company offers full-scale marketing research services, owned approximately 260,000 voluntarily registered samples across Japan, and had detailed sample sociodemographic data. Thus, the company was able to select specific sociodemographic group(s) from registered population according to requirements of each survey ordered. The participants in the present study were stratified into five age brackets (20–29, 30–39, 40–49, 50–59, and 60–69 years) and allocated equally to five sample groups (for each group, n = 200). Potential respondents (n = 4,068) were randomly and blindly invited to participate in the internet-based survey via e-mail. The invitation e-mail contained an URL directing them to a protected area of the website using their unique log-on ID and password. After 200 participants in each group voluntarily clicked on the “agree” button at the end of an online informed consent form and completed the questionnaire, acceptance of further participants was closed for each group. As an incentive for participation, the internet research service company offered reward points valued at 120 yen (one US dollar was equivalent to approximately 90 yen in 2009). The response rate was 24.6% (1,000/4,068). The study was approved by the Research Ethics Committee, Waseda University, Japan.

### Knowledge of breast cancer

Knowledge of breast cancer was measured by a 31-item questionnaire consisting of three subsections regarding symptoms (14 items), risk factors (13 items), and a screening test (4 items) for breast cancer. For each item, the participants responded by answering either true (1) or false (0). This questionnaire was adapted from previous studies of knowledge and attitudes about cancer [14,15,17]. The total score for each part were summed up the number of correct responses, except for the item about physical activity in risk factors subsection. Knowledge of breast cancer was then dichotomized into high and low scoring groups according to the median of each subsection. Individuals who responded ‘true’ to the statement that “engagement in no or low amounts of physical activity is a risk factor for breast cancer” were classified as being aware of the role of physical activity in breast cancer prevention.

### Sociodemographic variables

Age was classified according to five categories: 20–29, 30–39, 40–49, 50–59 and 60–69 years. Marital status was categorized as either currently married or unmarried. Having children was categorized into having children or no child. Education level was classified as high school graduate or less, junior college graduate or equivalent, and college graduate or higher. Employment status was categorized as working full time or not. Household income was classified into five categories: less than 3 million yen, 3–5 million yen, 5–7 million yen, 7–10 million yen and 10 million yen or more. Personal or familial history of breast cancer was categorized as having a history of breast cancer or not. Menopause status was categorized as postmenopausal or not.

### Exposure to information about physical activity and cancer

Respondents were asked about previous exposure to information about physical activity and cancer. They were categorized into two groups: ‘exposure to information’ or ‘no exposure to information’.

### Physical activity

Physical activity was assessed with a short version of the International Physical Activity Questionnaire (IPAQ-SV). The IPAQ-SV was designed to identify the frequency and duration of walking, moderate physical activity, vigorous physical activity, and sitting time in the previous week. The Japanese version of the IPAQ-SV has been observed to have acceptable validity and reliability [18]. Total physical activity was estimated by adding together the minutes per week of vigorous physical activity, moderate physical activity, and walking. Based on the physical activity recommendations of the American College of Sports Medicine and the American Heart Association, respondents were classified into three groups. Individuals who reported less than 10 minutes of total physical activity were categorized as “inactive”. Those who reported engaging in total physical activity from 10 to 149 minutes/week were denoted as “insufficiently active”, and those reporting 150 minutes/week or more activities were coded as “sufficiently active” [19].

### Data analysis

Data were analyzed for 985 adults who provided complete information for the study variable. Fifteen respondents with missing data were excluded from analyses. Chi-square tests were performed to examine proportional differences between sociodemographic characteristics and the awareness of the role of physical activity in breast cancer prevention. Moreover, forced-entry logistic regression analysis was conducted to examine the independent relationships between sociodemographic variables, knowledge, exposure to information, level of physical activity and awareness of the role of physical activity in breast cancer prevention adjusted for all independent variables. Adjusted Odds Ratios (AORs) and their 95% confidence intervals (CIs) were calculated. Statistical analyses were performed using the SPSS for Windows version 21.0 J for Windows (Statistical package for the Social Sciences; SPSS Inc. Chicago, IL, USA).

## Results

### Characteristics of participants

Table 1 presents sociodemographic characteristics of the study participants. The mean age (SD) was 44.3 (13.4) years, 74.3% were married, 35.2% had graduated from 4 years university or higher, and 29.7% were employed full time. Overall, 17.1% of the respondents had a household income of less than 3,000,000 yen whereas 13.3% earned more than 10,000,000 yen per year. Moreover, 34.8% were postmenopausal, and 5.1% reported a personal or familial history of breast cancer. Significant proportional differences in awareness of the role of physical activity in breast cancer prevention were observed. Those with children were less likely to be aware than those without a child (p = .002). Awareness was higher in those having greater educational attainment (p = .004).

**Table 1:** Sociodemographic characteristics and prevalence of awareness about the role of physical activity in breast cancer prevention

	**n**	**Sample %**	**% of individuals aware of the role of physical activity in breast cancer prevention**
**Full sample**	985	100.0	31.5
**Age/years**			
20–29	197	20.0	38.1
30–39	197	20.0	32.5
40–49	198	20.1	28.3
50–59	195	19.8	32.8
60–69	198	20.1	25.8
**Marital status**			
Unmarried	253	25.7	30.5
Married	732	74.3	34.4
**Having children**			
No	361	36.6	37.4
Yes	624	63.4	28.0
**Education**			
Less than high school graduate	303	30.8	25.7
2 years college or equivalent	335	34.0	30.1
College graduate	347	35.2	37.8
**Employment status**			
Not full time	692	70.3	30.2
Employment full time	293	29.7	34.5
**Household income/yen**			
<3,000,000	168	17.1	36.3
<5,000,000	266	27.0	28.9
<7,000,000	237	24.1	29.5
<10,000,00	183	18.6	33.3
≥10,000,000	131	13.3	31.3

The median of knowledge score about breast cancer symptoms was 10.2 (out of 14), and 55.6% of the respondents were classified as belonging to the high level of knowledge group on this basis. The median of knowledge score about breast cancer risk factors was 6.8 (out of 12), and 58.6% were classified as belonging to the high level group. The median knowledge score about breast cancer screening was 3.8 (out of 4), and 82.0% of participants were classified as belonging to the high level group. More than 60 percent (62.7%) of the participants reported that they were exposed to information about physical activity and cancer from some information source. Overall, 57.4% of the respondents were sufficiently active, whereas 24.3% were inactive.

### Awareness and correlates of awareness the role of physical activity in breast cancer prevention

The prevalence of awareness about the preventive role of physical activity in breast cancer among Japanese women was 31.5% (95% CI: 28.6-34.4). Table 2 presents the results of forced-entry regression analysis. Being married (AOR = 1.75; 95% CI: 1.05–2.92) was positively related to awareness of the role of physical activity in breast cancer prevention. Having children (AOR = 0.65; 95% CI: 0.36–0.86) was negatively related to awareness of the role of physical activity in breast cancer prevention. In addition, those with 4 year university degrees or higher (AOR = 1.50; 95% CI: 1.01–2.22) were significantly more likely to be aware of the preventive effect of physical activity on breast cancer than those with less than high school graduate levels of education. Moreover, exposure to information (AOR = 2.11; 95% CI: 1.51–2.95) and high knowledge of breast cancer symptoms (AOR = 2.43; 95% CI: 1.75–3.36) were positively associated with awareness of the role of physical activity in breast cancer prevention. Finally, low knowledge of breast cancer risk factors (AOR = 0.30; 95% CI: 0.22–0.40) was negatively associated with awareness of the role of physical activity in breast cancer prevention.

**Table 2:** Multivariate logistic models for awareness of the role of physical activity in breast cancer prevention

	**n**	**OR**	**(95% CI)**	**AOR**	**(95% CI)**
**Age**					
20–29	197	1	(ref)	1	(ref)
30–39	197	0.78	(0.52–1.36)	0.79	(0.36–1.73)
40–49	198	0.64	(0.42-0.98)**	1.15	(0.59–2.25)
50–59	195	0.80	(0.53-1.20)	0.62	(0.37–1.05)
60–69	198	0.56	(0.37-087)*	0.91	(0.56–1.49)
**Marital status**					
Unmarried	253	1	(ref)	1	(ref)
Married	732	0.84	(0.62-1.13)	1.75	(1.05–2.92)*
**Having children**					
No	361	1	(ref)	1	(ref)
Yes	624	0.65	(0.50-0.86)**	0.56	(0.36–0.86)**
**Education**					
Less than high school graduate	303	1	(ref)	1	(ref)
2 years college or equivalent	335	1.25	(0.88-1.76)	1.00	(0.68–1.49)
College graduate	347	1.75	(1.25-2.45)**	1.50	(1.01–2.22)*
**Employment status**					
Not full time	692	1	(ref)	1	(ref)
Employment full time	293	1.22	(0.99-1.63)	1.04	(0.72–1.50)
**Household income/yen**					
<3,000,000	168	1	(ref)	1	(ref)
<5,000,000	266	0.72	(0.47-1.08)	0.65	(0.40–1.04)
<7,000,000	237	0.74	(0.48-1.12)	0.58	(0.40–1.08)
<10,000,00	183	0.88	(0.57-1.36)	0.79	(0.47–1.33)
≥10,000,000	131	0.80	(0.49-1.30)	0.66	(0.37–1.17)
**Menopause**					
Pre-menopause	642	1	(ref)	1	(ref)
Post-menopause	343	0.71	(0.53-0.95)	0.62	(0.34–1.11)
**Cancer history**					
No	935	1	(ref)	1	(ref)
Yes	50	1.36	(0.75-2.44)	0.95	(0.50–1.82)
	n	OR	(95%CI)	AOR	(95%CI)
**Exposure to information**					
No	267	1	(ref)***	1	(ref)
Yes	618	2.51	(1.85-3.39)***	2.11	(1.51–2.95)***
**Knowledge of breast cancer (symptoms)**					
Low	437	1	(ref)	1	(ref)
High	548	2.48	(1.86-3.30)***	2.43	(1.75–3.36)***
**Knowledge of breast cancer (risk factor)**					
Low	408	1	(ref)	1	(ref)
High	577	0.28	(0.21-0.38)***	0.30	(0.22–0.40)***
**Knowledge of breast cancer (screening test)**					
Low	177	1	(ref)	1	(ref)
High	808	1.43	(0.99-2.07)	1.16	(0.76–1.76)
**Level of physical activity**					
Inactive	565	1	(ref)	1	(ref)
Some regular activity	181	1.23	(0.80-1.88)	0.98	(0.61–1.59)
Meet physical activity recommendations	239	1.39	(0.99-1.94)	1.21	(0.83–1.77)

Notes. OR, odds ratio; AOR, adjusted odds ratio; CI, confidence interval.; ref, referent group. *p < .05; **p<.01; ***p < .001.

Note: The multivariate model examined all variables simultaneously and mutually adjusted them for each other.

## Discussion

This is the first study to identify the prevalence and correlates of awareness for the preventive effect of physical activity on breast cancer among adult women through internet-based study. The prevalence of awareness was approximately 30%. Compared with previous studies examining colon cancer across 21 European countries (30%) [15] and in the United States (15%) [14], the awareness level in the present study was similar or somewhat higher. It may be that the similarities and differences in awareness with other studies may partly due to differences in methodology, and the use of structured or open-ended questions [20]. However, the results of this study and others [14,15] generally indicate a low level of awareness about the role of physical activity in cancer prevention regardless of country or type of cancer. In Japan, the Cancer Control Act which indicates the need to inform individuals about the effect of lifestyle including physical activity on health and cancer prevention [21] was implemented in 2007. The low awareness found among Japanese women in this study suggests that information about the role of physical activity in breast cancer prevention may not have been widely disseminated yet. The Health Information National Trend Survey in 2003 revealed that almost 70% of Americans adults who believed that physical activity could help to prevent cancer did not specify which specific type(s) of cancer it helps to prevent [22]. Thus, it is apparently necessary to develop effective health communication strategies that aim to raise community-level awareness about the positive relationship between breast cancer prevention and physical activity.

The present study found several sociodemographic factors related to awareness of the role of physical activity in breast cancer prevention. Individuals having children were less likely to be aware of this role. The result imply that women having children may be unable to take enough time and access information because they spend more time on child care than do men [23]. On the other hand, marriage, and having a high level of education were found to be positively associate with awareness. Several studies have indicated that marital status is a consistent correlate of breast cancer knowledge and screening behavior [24,25]. With regard to education level, the present finding is consistent with results from previous research that reported a positive association between education level and cancer-related knowledge [26,27]. Further research is warranted to further clarify the sociodemographic correlates of awareness about the positive relationship between breast cancer and physical activity or other protective behaviors.

Respondents who had greater knowledge about breast cancer risk factors had lower awareness of the connection to physical activity than respondents with less knowledge. The result suggests that the role of physical activity in breast cancer prevention may not have been disseminated widely enough, even if individuals had high knowledge about breast cancer risk. In contrast, respondents who had previous exposure to information and higher knowledge about breast cancer symptoms had higher awareness than their counterparts with less exposure and less knowledge of symptoms. These findings may indicate that providing clearer cancer information and, more specifically, information about the effect of physical activity in reducing cancer risk may be necessary for increasing awareness of the role of physical activity in breast cancer prevention. Jorgensen, Wayman, Green, and Gelb [28] suggested that the effectiveness of the mass media in conveying information can play an important role in the public understanding information and knowledge about health and cancer. Thus, utilization of health communication approaches such as mass media campaigns through which information about cancer and cancer prevention could be widely received to the public should be prioritized. Previous studies mentioned that vulnerable groups that lack an interest in health, or cannot afford to have interest are hard to receive a benefit of the approach [29,30]. Thus, it may also be necessary to select more effective strategies tailored to reaching groups with lower awareness such as those with lower education and having children and unmarried women. For example, an effective approach to improving awareness may be to use cancer screening and cancer screening coupon distribution based on guidelines, medical examination, and events for children.

The present study did not find an association between awareness and the level of physical activity engaged in by the participants. However, this finding is consistent with a previous study that found that mass media campaigns increased awareness and knowledge of physical activity, but had less impact on changes in physical activity levels [31]. Previous research indicated that the influence of media campaigns related to healthy diet and physical activity was limited to awareness and attitudes [32]. Nevertheless, awareness is considered an essential first step in changing physical activity behavior [11]. Therefore, it may be especially important to make an effort to increase women’s awareness of the role of physical activity in breast cancer prevention. In addition, it would be necessary to design segmented information campaigns for people who respond differently to information. Regular physical activity and exercise habits are effective not only for breast cancer prevention but also for the prevention of other diseases. Thus there may be women, for example, without awareness of the effective role of physical activity in breast cancer prevention, but who take regular physical activity or exercise.

The present study has several limitations. First, these data were cross-sectional, thereby making it impossible to determine cause and effect. Second, the level of physical activity was assessed using only the self-reported questionnaire. Thus, inaccurate estimation of physical activity level and recall bias were unavoidable. Moreover, the outcome variable for this study—awareness of the role of physical activity in breast cancer prevention—was measured using a single item. Although it was adapted from previous studies [14,15,17], other measurement approaches may yield varying estimates of awareness. Finally, the present study was conducted via the internet. Selection bias, because of the non-representative nature of the internet population and the self-selection of participants, is a major factor limiting the generalizability of results in the study [33]. However, random selection of participants, an equally distributed age group between 20–69 years, from a pool of 260,000 people may have helped to mitigate bias and ensure greater representativeness in the survey.

## Conclusions

Japanese women through internet based study were poorly aware of the role of physical activity in breast cancer prevention. Awareness was especially low among individuals with children and higher knowledge of breast cancer risk factors whereas high in married women, those with higher educational level, exposure to information about physical activity and cancer, and higher knowledge of breast cancer symptoms. The findings suggest that the development of communication strategies to increase the awareness of the preventive role of physical activity on breast cancer is needed for cancer prevention. Further identification of the specific psychological and behavioral characteristics of subgroups with low awareness would help in developing more effective and targeted communication strategies.

## Competing interests

The authors declare that they have no competing interests.

## Authors’ contributions

RM was involved in the design of the study, analysis and interpretation of the data, and drafting the manuscript. AS, KI, and KO involved in the design and coordination of the study and helped in drafting the manuscript. All authors read and approved the final manuscript.

## Pre-publication history

The pre-publication history for this paper can be accessed here:

http://www.biomedcentral.com/1472-6874/14/80/prepub
